# Transcranial direct current stimulation over motor cortex improves pain in end stage renal disease a randomized controlled trial

**DOI:** 10.1038/s41598-025-96415-7

**Published:** 2025-07-01

**Authors:** Eduarda Fonseca Mendes, Edson Silva-Filho, Joanna Brito Holanda, M. G. L. Matias, Tatiana Camila Lima, Sara Costa, Artur Quintiliano, Kevin Pacheco-Barrios, Felipe Fregni, Rodrigo Pegado

**Affiliations:** 1 Graduate Program in Heath Science, Federal University of Rio Grande do Norte, 620 Nilo Peçanha St., Natal, Rio Grande do Norte 59012-300 Brazil; 2 Graduate Program in Physical Therapy, Federal University of Rio Grande do Norte, Natal, Rio Grande do Norte Brazil; 3 Department of Medicine, Federal University of Rio Grande do Norte, Natal, Rio Grande do Norte Brazil; 4 Nephrology and Hypertension, Mayo Clinic, Rochester, MN USA; 5 Neuromodulation Center, Spaulding Rehabilitation Hospital, Massachusetts General Hospital, Harvard Medical School, Boston, MA USA; 6 Universidad San Ignacio de Loyola, Vicerrectorado de Investigación, Unidad de Investigación para la Generación y Síntesis de Evidencias en Salud, Lima, Peru

**Keywords:** Chronic pain, Rehabilitation, Neuromodulation, Noninvasive brain stimulation, Hemodialysis, Neuroscience, Neurological disorders

## Abstract

Improving pain management and physical function remains a significant challenge for patients affected by end-stage renal disease (ESRD) undergoing hemodialysis. Non-pharmacological interventions, such as transcranial direct current stimulation (tDCS), have gained attention for their potential to alleviate pain and improve functional outcomes. This study explored the effects of tDCS on pain, fatigue, and functional performance in patients affected by ESRD undergoing hemodialysis and experiencing chronic pain. Thirty-four participants were randomized to receive either active or sham tDCS, with 15 sessions administered over five weeks. Anodal tDCS over C3 with an intensity of 2 mA was administered in 15 sessions, 3 times per week. Visual Analogue Scale for pain (VAS), fatigue, functional performance (sit-to-stand test, stationary walk, timed up and go test (TUG), elbow flexion test, and manual dynamometry) were assessed. We found a significant difference in VAS (*p* < 0.001) between groups and across time (fifteenth session *p* < 0.001; at the first, *p* < 0.001; and second follow-up, *p* < 0.001). TUG showed significant differences between-group analysis (*p* = 0.009). No significant differences between groups were observed for fatigue (*p* = 0.21) and all others functional tests. These findings suggest that tDCS may be a promising, non-invasive intervention for managing pain and improving mobility in patients affected by ESRD undergoing hemodialysis. Future studies with larger samples and extended follow-ups are warranted to further validate these findings and explore the long-term benefits of tDCS. Trial registration: The study is registered on the Brazilian Clinical Trials Registry (ID: RBR-7kym6v8).

## Introduction

Chronic kidney disease (CKD) is a growing global health problem affecting more than 10% of the work population and continuously increase as one of the leading causes of death and disability worldwide^1,2^. Patients affected by CKD evolve with poor quality of life, low physical function and chronic pain, specifically among patients with end-stage renal disease (ESRD)^3^. The prevalence of chronic pain in patients affected by ESRD undergoing hemodialysis is 60.5% and the mean prevalence of moderate or severe pain is 43.6%^4^. Even with a high prevalence of chronic pain in ESRD, the pain management strategies have shown limited evidence and are based on indirect pharmacological evidence and clinical expertise^3,4^.

Chronic pain in patients affected by ESRD is multifactorial; often associated with peripheral polyneuropathy, musculoskeletal issues, peripheral vascular disease, polycystic kidney disease, malignancy, and calciphylaxis^5^. It involves nociceptive, neuropathic, and inflammatory process according to the etiology^3^. Chronic pain negatively impacts cognitive and physical functions, disrupting daily and social life, and it is associated with an increased incidence of depression, sleep disturbances, low mood, attention deficits, and impairment in everyday functioning^3^.

Due to the nature of chronic pain in patients affected by ESRD, maladaptive mechanisms likely occur in the peripheral and central nervous systems, resulting in imbalanced neural compensatory mechanisms^6,7^. Maladaptive changes in the pain control system are associated with a reduction in inhibitory brain activity, leading to impaired pain control^8^. Regions of the pain modulatory system, which are associated with pain perception, cognitive control of emotion, and self-referential processing, may be defective in these patients^9^. Several brain disorders have been demonstrated in ESRD, with significant changes and decreased cognitive functions, poor mood states, and impaired pain control modulation^10–12^. Pain management in patients affected by ESRD is often ineffective, and the availability of management resources, including access to non-pharmacological approaches, needs to be investigated^13^.

Neuromodulation techniques could be a valuable non-pharmacological, well-tolerated, and safe adjunct therapy to improve pain in this population due to induced alterations in the sensory-motor brain networks^14,15^. Transcranial direct current stimulation (tDCS) was generally administered for chronic pain and has shown moderate efficacy in several pain conditions^16,17^. The anodal tDCS over the primary motor cortex and the cathodal positioned over the contralateral supraorbital area modulates motor cortex excitability and influences other brain regions, such as anterior cingulate and insular cortex, thalamus and cerebellum^18^. This modulation enhances the activation of the endogenous pain modulation system, thereby improving the control of perceived pain intensity^19,20^. It is important to note that the tDCS-plasticity-induced depends on multiple factors, including the intensity, time of stimulation, and montage^21,22^. The use of tDCS may improve imbalanced neural activity and improve pain in several chronic pain conditions including fibromyalgia, neuropathic pain, and musculoskeletal pain^15,23^. TDCS is usually used to improve pain but could influence other aspects including mood, quality of life and physical function^24^. Recent findings using tDCS have demonstrated significant pain relief in patients with ESRD following ten non-consecutive sessions of 2 mA, 20-minute anodal tDCS over the left motor cortex^14^.

Given these initial findings, there is a need to explore the long-term effects of tDCS by increasing the number of sessions and investigating new outcomes in patients affected by ESRD. Additional sessions can promote long-term effects by strengthening the modulation of neurotransmitters, promoting biochemical adaptations, and influencing inflammatory markers, thereby amplifying therapeutic outcomes^25^. This study aims to evaluate the effects of anodal tDCS over the left primary motor cortex on pain (primary outcome), functional performance, and fatigue in patients affected by ESRD following an extended stimulation protocol.

## Results

Thirty-four individuals participated in this trial. Table 1 illustrates the clinical and sociodemographic data of the two groups. Most participants were female, married, and had an education level up to elementary school. The average age was over fifty years. The majority of patients had been undergoing hemodialysis for up to five years and had hypertension as the underlying cause of their end-stage renal disease. No statistical differences were found between the groups for clinical and sociodemographic data at baseline.

**Table 1:** Illustration of the clinical and sociodemographic data from active and Sham groups.

Variables	Active tDCS (*n* = 17)	Sham tDCS (*n* = 17)
Age^a^	52 ± 17.9	55.4 ± 17.1
Female sex^b^	58.8	58.8
BMI^a^	25.5 ± 3.94	26.8 ± 8.40
Marital status^b^		
Married	26.5	20.6
Single	14.7	14.7
Divorced	5.9	2.9
Widow	2.9	11.8
Education level^b^		
Illiterate	8.8	0
Elementary school	26.5	38.2
High school	14.7	11.8
Smoke (yes)^b^	50	50
Hemodialysis time^b^		
1 to 5 years	32.4	35.3
6 to 10 years	8.8	11.8
11 to 16 years	5.9	0
> 17 years	2.9	2.9
Vascular access to hemodialysis^b^		
Arteriovenous fistula	50	41.2
Long-term catheter	0	5.9
Permcath	0	2.9
Fractures (No)^b^	44.1	47.1
Etiology^b^	23.6	8.9
Hypertension	0	8.8
Diabetes	2.9	5.9
Hypertension and diabetes	8.8	2.9
Glomerulonephritis	11.8	14.7
Nephropathy	0	2.9
Neurogenic bladder	2.9	5.9

*BMI* body mass index. ^a^Mean and standard deviation. ^b^Percentage.

**Fig. 1:**
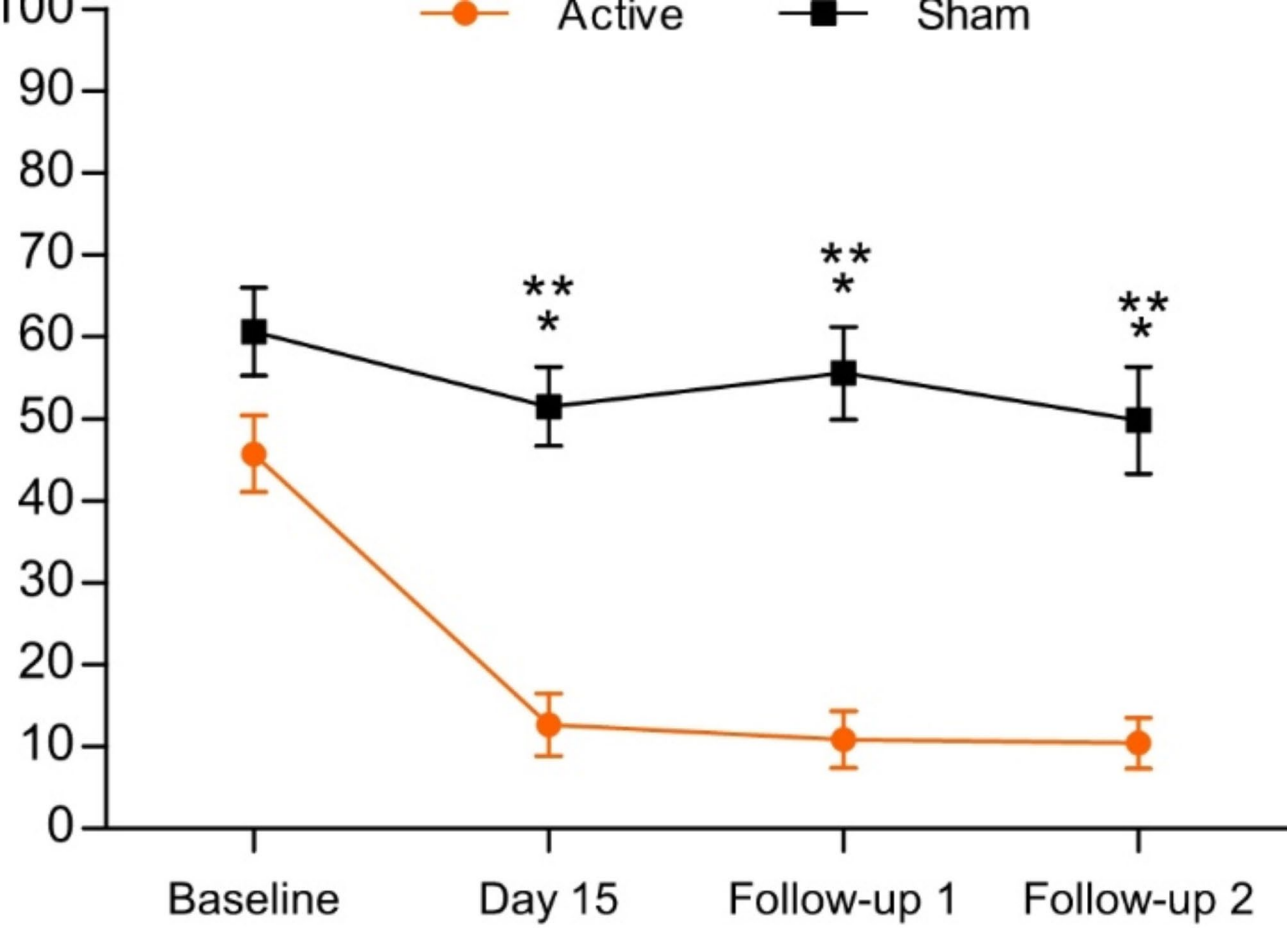
Mean visual analogue scale (VAS) pain intensity before treatment (baseline), on day 15 (after the last session) and follow-ups. Error bars denote standard error of the mean. *Denote significant difference between groups for each day. **Denote significant difference between baseline only for active group.

A significant difference in VAS scores was found between the groups for four time point analysis (*p* < 0.001) (baseline, day 15 and two follow-ups) (Fig. 1). Intergroup analysis showed a significant difference on day 15 (*p* < 0.001), first follow-up (*p* < 0.001) and second follow-up (*p* < 0.001) (Fig. 1). Intragroup analysis showed a significant difference between baseline with day 15 (MD: 33.05; *p* < 0.001), first follow-up (MD: 34.88; *p* < 0.001) and second follow-up (MD: 35.29; *p* < 0.001) (Fig. 1). No difference was found for sham group.

**Fig. 2:**
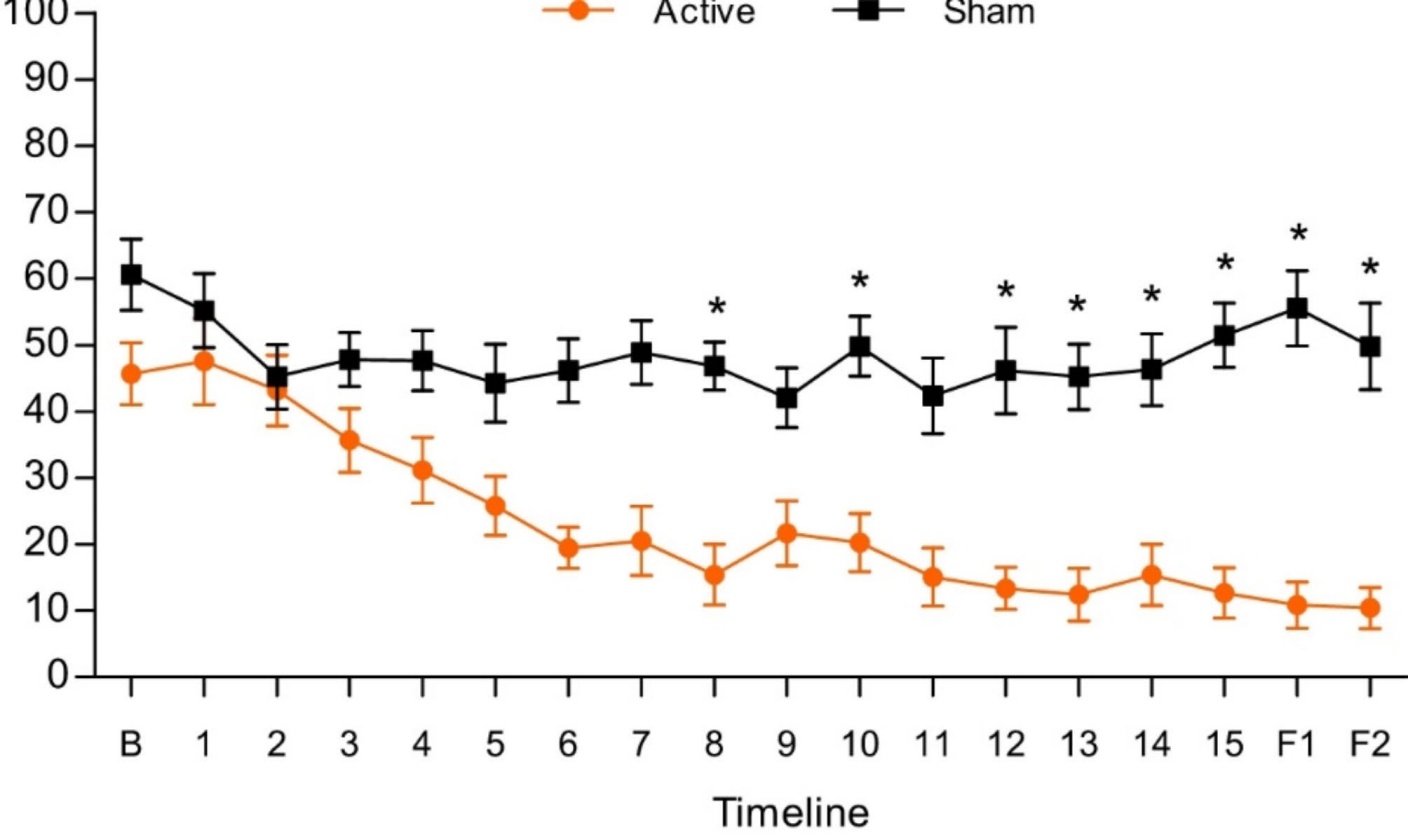
*Between-group difference for visual analogue scale (VAS) pain intensity for each day. B: Baseline; 1 to 15: tDCS sessions; F1: first follow-up; F2: second follow-up.

A second analysis using daily access of VAS showed a significant difference between groups (*p* < 0.001) (Fig. 2). The post-test showed a significant difference between groups for VAS at session 8 (MD: -31.41. [CI 95%: -45.34, -17.48]; *p* = 0.009), session 10 (MD: -29.52; [CI 95%: -43.45, -15.59]; *p* = 0.02), session 12 (MD: -32.82; [CI 95%: -46.75, -18.89]; *p* = 0.004), session 13 (MD: -32.82; [CI 95%: -46.75, -18.89]; *p* = 0.004), session 14 (MD: -30.88; [CI 95%: -44.81, -16.95]; *p* = 0.01), session 15 (MD: -38.82; [CI 95%: -52.75, -24.89]; *p* < 0.001), first follow-up (MD: -44.70; [CI 95%: -58.63, -30.77]; *p* < 0.001), and second follow-up (MD: -39.35; [CI 95%: -53.28, -25.42]; *p* < 0.001). It is important to note that session 4 (MD: -16.52 [-29.93, -3.11]), session 5 (MD: -18.52 [-31.93, -5.11]), session 6 (MD: -26.70 [-40.11, -13.29]), session 9 (MD: -20.41 [-33.82, -6.00]), and session 11 (MD: -27.29 [-40.70, -13.88]) showed p-values greater than 0.05. However, the confidence interval between the groups did not cross zero.

Table 2 illustrates the fatigue and functional tests measured between the groups. No significant differences between the groups were observed for fatigue (*p* = 0.21). Intra-group analysis demonstrated a significant reduction in fatigue at the first follow-up (MD: 17; *p* < 0.001) and the second follow-up (MD: 17.64; *p* < 0.001) compared to baseline, specifically in the active group. No intra-group significant difference was found for sham. The Patient Global Impression of Change scale showed no statistical difference.

**Table 2:** Secondary outcomes comparisons before and after intervention for the study groups.

Functional test	Active tDCS	Sham tDCS	Between-group difference
Fatigue			10.88 (5.22;16.53)
Baseline	37.6 (31.9;43.3)	43.6 (37.9;49.3)	
1st follow-up	20.6 (14.9;26.3)	35.2 (29.5;40.9)	
2nd follow-up	19.9 (14.2;25.7)	32.0 (26.1;37.9)	
Within-group change	26.03 (20.30;31.76)	36.93 (31.14;42.72)	
Elbow flexion test^a^			-1.70 (-4.66;1.26)
Baseline	7.29 (4.83;9.76)	6.59 (4.13;9.05)	
1st follow-up	10.18 (7.72;12.64)	9.29 (6.83;11.76)	
2nd follow-up	13.12 (10.66;15.58)	9.60 (7.11;12.10)	
Within-group change	10.20 (7.73;12.67)	8.49 (6.02;10.96)	
Timed up and go^a^			2.12 (0.63;3.61)*
Baseline	10.73 (9.52;11.93)	11.98 (10.77;13.18)	
1st follow-up	8.49 (7.29;9.70)	11.62 (10.42;12.83)	
2nd follow-up	9.55 (8.35;10.76)	11.55 (10.33;12.77)	
Within-group change	9.59 (8.38;10.80)	11.72 (10.51;12.93)	
Stationary walk			-3.81 (-10.91;3.28)
Baseline	17.1 (11.2;23.1)	19.4 (13.4;25.3)	
1st follow-up	26.2 (20.3;32.2)	24.4 (18.5;30.4)	
2nd follow-up	36.1 (30.1;42.0)	24.2 (18.2;30.2)	
Within-group change	26.47 (20.55;32.39)	22.67 (16.71;28.63)	
Manual dynamometer			-3.22 (-9.38;2.94)
Baseline	19.6 (14.9;24.3)	16.0 (11.4;20.7)	
1st follow-up	22.0 (17.3;26.6)	19.2 (14.6;23.9)	
2nd follow-up	23.1 (18.4;27.7)	19.7 (15.0;24.4)	
Within-group change	21.23 (18.53;23.93)	18.3 (15.60;21.0)	

^a^Data expressed as mean (95% confidence interval). Between-group difference: sham tDCS group minus active tDCS group.

Among the functional tests, GEE revealed a statistically significant difference only for the Timed Up and Go (*p* = 0.009). However, the confidence intervals did not cross zero for sit-to-stand (MD: 1.88 [0.31, 3.44]), elbow flexion (MD: 3.51 [0.08, 6.94]) and stationary walk (MD: 11.85 [3.55, 20.15]) (Table 2).

No serious adverse effects or clinically relevant hemodynamic changes were reported by study participants during the trial. Side effects such as itching and tingling were reported in both groups, while skin redness and burning sensation were observed only in the active tDCS group (Table 3). The active group presented 82% of responders, while the sham group presented 58%, considering a minimal clinically important difference > 20 mm for VAS after the 15th session, 1st or 2nd follow-up. There were no differences between the groups after the 15th session for patient global impression of change scale (χ^2^ = 2.69; *p* = 0.84).

**Table 3:** Adverse effects reported by participants in both study groups.

Adverse effect	Active tDCS (*n* = 17)	Sham tDCS (*n* = 17)
Skin redness	20%	0%
Headache	0%	0%
Dizziness	0%	0%
Itching	60%	66%
Tingling	10%	33%
Neck pain	0%	0%
Burning sensation	10%	0%

At the end of the study, we asked the participants to guess whether they had received active or sham tDCS. No significant differences were observed between groups in their ability to correctly identify their allocation (*p* = 1.00).

## Discussion

This study demonstrates the efficacy of tDCS to improve pain and functional mobility in patients affected by ESRD undergoing hemodialysis. While clinical effects on pain were noticeable from the fourth session, statistically significant results were observed starting from the eighth session and persisted until the one-month follow-up. So, it seems that tDCS may be considered a strategy to reduce pain and improve functional performance of patients with end-stage renal disease.

The use of non-invasive brain stimulation to reduce pain has been extensively investigated over the years. tDCS is one of the non-invasive techniques that has shown efficacy in treating various pain-related conditions^16,26^. TDCS modulates different structures within the central nervous system, including neurotransmitters^27^, blood flow^28^, soma, and axons^29^. Due to the chronic pain stimuli disrupting normal pain processing within the central nervous system, different tDCS montages involving different brain areas have been tested to assess central nervous system responsiveness^30,31^. The brain areas, collectively known as the pain neuromatrix, involve the dorsolateral prefrontal cortex, primary motor cortex, sensory cortex, thalamus, cerebellum and supplementary motor area, which are responsible for pain processing^32^. In this context, tDCS applied to the primary motor cortex appears to be the most effective target for improving central nervous system function and reducing pain^30^. In this study, tDCS montage involved anodal stimulation over the left primary motor cortex with 2 mA for 20 min was based on a previous study with ESRD^14^. It is important to highlight that our trial revealed significant reductions in VAS pain scores, with mean differences of -38.82, -44.70, and − 39.35 at session 15, first follow-up, and second follow-up, respectively. These findings are particularly noteworthy given the established minimal clinically important difference for VAS pain, which is typically around 10–20 mm^26,33^. Our results not only surpass this threshold but demonstrate a robust and clinically meaningful improvement in pain, achieved with very minor side effects such as temporary tingling and itching sensations. This underscores the potential of tDCS as an effective and well-tolerated intervention in this vulnerable population.

A significant number of patients with end-stage renal disease experience chronic pain, including severe pain^4^. The pain experienced by patients affected by ESRD is multifaceted, arising from various etiologies and involving comorbidities, limited functional mobility due to skeletal muscle problems, and disrupted individual pain thresholds^34^. Surprisingly, we observed statistical improvement in the timed up and go test after stimulation. Given the short duration of stimulation, we did not expect to find significant improvements in motor performance. However, it is possible that the reduction in pain influenced motor function, leading to better functional performance^35^ due to an improvement in sensorimotor integration and reduction of kinesiophobia.

Fatigue scores significantly decreased following the interventions, although no different between groups. This reduction in fatigue may be attributed to the involvement of central fatigue which presents association with the emergence and maintenance of task functions. Stimulation over the primary motor cortex has been associated with enhancements in corticomuscular coherence and neural pathways, thereby improving cortical-muscular functional coupling and motor control performance^36^. Additionally, as corticospinal excitability declines with fatigue, improvements in task performance may also be linked to addressing intracortical dysfunction^37^. Thus, tDCS might be a viable option for reducing fatigue in patients with ESRD. The effects observed after 15 non-consecutive sessions were modest and insufficient to demonstrate between-group differences in fatigue following the treatment and follow-up.

It is important to mention that this is the second trial that evaluated the effects of tDCS on pain, functional performance, and fatigue in ESRD patients. The first trial designed implemented 10 non-consecutive sessions in 15 patients per group^14^. Notably, the authors found clinically significant results at the 10th session that remained for one-month, mean difference 3.13 and 2.6, respectively for numeric rating scale. We found in this trial a similar improvement that also lasted for one month. These preliminary results must stimulate further research by increasing the number of sessions and longer follow-up in this population.

This study has some limitations that must be acknowledged. The small sample size limited our ability to identify statistical significance in certain analyses. For pain, a p-value of less than 5% was observed only when the differences between groups reached approximately 30 points due to the groups’ variability. Additionally, a longer follow-up period could better demonstrate the long-term effects of the 15 sessions. One of the limitations of this study is the lack of detailed characterization of pain etiology and location, which are clinically relevant factors that may influence treatment outcomes. Although randomization was employed to balance potential confounders, the absence of this information limits the ability to determine whether differences in pain characteristics existed between groups. Data analysis was conducted with full transparency, carefully considering the smaller sample size and its potential implications. Nonetheless, given the observed effect size and its clinical significance, the results provide valuable contributions to the literature and may inform clinical practice. However, it is important to note that additional studies are necessary to replicate and expand upon these findings.

This study demonstrated that 15 non-consecutive sessions of anodal tDCS over the left primary motor cortex improved pain and functional mobility in patients affected by end-stage renal disease. tDCS emerges as a promising new strategy for addressing the often-neglected pain symptomatology in patients undergoing hemodialysis.

## Materials and methods

### Study design

A double-blind, two-arm, parallel, randomized, sham-controlled trial was conducted between October 2023 and April 2024 at the Kidney Institute in Natal, Brazil. The trial adhered to the resolution No. 466/12 of the National Health Council, the Declaration of Helsinki, and CONSORT 2010 guidelines^38^. The study received approval from the Federal University of Rio Grande do Norte Ethical Committee (registration number 5.743.008) and is registered on the Brazilian Clinical Trials Registry under code RBR-7kym6v8 (Date of registration: 22/08/2023). Patients undergoing hemodialysis were invited to participate in the study after being informed about the procedures. Those who agreed signed the informed consent form.

### Participants

We included patients who met the following criteria: (1) men or women with a minimum age of 18 years; (2) undergoing hemodialysis for chronic kidney disease stage 5D for more than 3 months during 4-hour sessions; (3) experiencing chronic pain (chronic musculoskeletal pain, chronic headache, and/or chronic neuropathic pain) with a visual analog scale score of more than 4 (on a scale of 1 to 10) for over 3 months^39^; (4) understanding the study explanations and questionnaires; (5) no electrical implants in the body; (6) no history of epilepsy or convulsions; (7) no metal implant in the scalp or brain; (8) no pregnancy; (9) no signs of severe disease or indications for hospitalization, including hemodynamic instability, infection, acute myocardial infarction, or stroke. Patients undergoing physical therapy, those with disorientation, or those with skin lesions in the stimulation area were excluded from the study.

### Interventions

A trained physical therapist, blinded to the assessment, administered a total of 15 tDCS sessions, conducted three times per week on non-consecutive days (Monday, Wednesday, and Friday, or Tuesday, Thursday, and Saturday). Each patient remained awake and rested in a comfortable chair with back and arm support during the tDCS intervention, which took place during their regular hemodialysis sessions. The tDCS device Microestim Genius was used for this study (NKL, Souza Cruz Brusque/SC, Brazil). The device delivered a monophasic continuous current at an intensity of 2 mA for 20 min. To initiate the intervention the physical therapist positioned the anode electrode over the left primary motor cortex (C3) and the cathode electrode over the right supraorbital region (Fp2), following the International 10–20 EEG system. The electrodes were positioned within 35 cm² sponges hydrated with saline solution (154 mM NaCl, approximately 12 mL per sponge) and secured to the scalp with an elastic band. After positioning the electrodes, a gradual 30-second current ramp-up was performed for stimulation. At the end of the tDCS session, a gradual 30-second ramp-down was performed. The sham tDCS group followed the same protocol, with a 30-second ramp-up at the beginning and a 30-second ramp-down at the end of the session, creating an initial and final sensation similar to active tDCS, but without any modulatory effect the resting 19 min. The device displays were identical in both active and sham settings^40^. For ethical reasons, we did not alter hemodialysis routines (including the days and locations of hemodialysis sessions), clinical care (medications, imaging, or blood tests), or prior prescriptions of analgesics or other medications.

### Outcomes

**Fig. 3:**
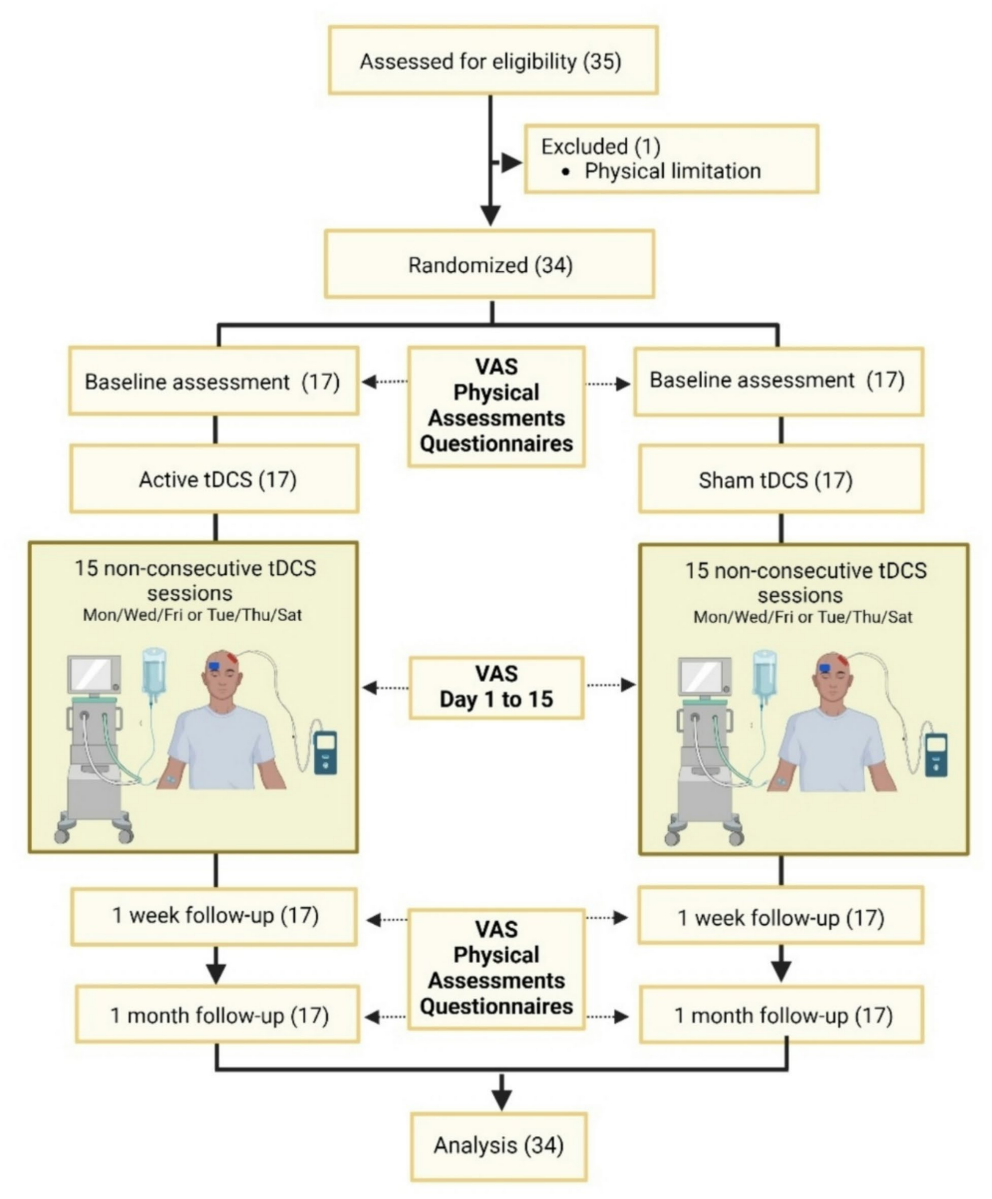
Flowchart of the study. *VAS* visual analogue scale, *tDCS* transcranial direct current stimulation, *Mon/Wed/Fri* Monday/Wednesday/Friday, *Tue/Thu/Sat* Tuesday/Thursday/Saturday.

A second physical therapist, blinded to the group allocation, conducted the assessments at all phases of the study. Initially, data collection was performed through sociodemographic information such as name, sex, age, weight, height, marital status, education level, hemodialysis time, vascular access to hemodialysis, fractures, and etiology. Pain was assessed using visual analogue scale (VAS) at baseline, after each of the 15 sessions, until one week after the 15th session (1st follow-up), and one month after the 15th session (2nd follow-up). Fatigue, functional performance, and patient global impression of change were assessed at baseline, at 1st follow-up and 2nd follow-up (Fig. 3).

Pain intensity measured on VAS was the primary outcome. VAS is a unidirectional measure of pain intensity widely used for diverse chronic pain condition^34^. This is a horizontal continuous scale with 100 mm in length anchored by “no pain” (score of 0) and “worst imaginable pain”. Participants were instructed to draw a perpendicular line to the VAS line to indicate their current level of pain. The score was determined by measuring the distance (in millimeters) between the “no pain” anchor and the mark made by the patient^41^.

Fatigue and functional performance were assessed as a secondary outcome. To access functional performance, it was used the sit-to-stand test, stationary walk, timed up and go test, elbow flexion test, and manual dynamometry.

Fatigue was assessed using the Fatigue Severity Scale, where patients self-reported their fatigue through 9 items scored on a scale ranging from 1 (strongly disagree) to 7 (strongly agree)^42^. This scale categorizes fatigue levels as follows: 0 to 28 indicates no fatigue; 29 to 39 indicates mild fatigue; 40 to 51 indicates moderate fatigue; and 52 to 63 indicates severe fatigue. The Fatigue Severity Scale demonstrates strong internal consistency of 0.88 and good test–retest reliability of 0.84^43^.

To assess lower limb strength, the 30-second sit-to-stand test was performed. During this test, patients were instructed to sit and stand as quickly as possible for 30 s with their arms crossed over their chest and their legs aligned with their shoulders^44^. To calculate the results, we measured the number of times they sat and stood for 30 s.

For the stationary walk test the patients were asked to perform a stationary walk for 2 min, during the test we counted the number of knee (one knee) lifts to assess their performance^45^.

Timed up and go test was used to evaluate the overall functional mobility. This assessment provides a dependable, cost-effective, safe, and time-efficient method to assess overall functional mobility^46^. For the timed up and go test, patients began seated in a chair. Upon receiving the command, they stood up, walked 2.5 m, and returned to the chair to sit down. The stopwatch was started when the researcher gave the command to begin and stopped when the patient sat down again^47^.

The elbow flexion test, performed on the dominant arm, measured upper limb function. Patients were asked to hold a 2-kg dumbbell and flex and extend their elbow as quickly as possible within 30 s^48^. To measure the result, we counted how many flexions the patient performed. The manual dynamometry test was conducted on the dominant upper limb using a portable dynamometer. The patients performed the task in a standing position, with their shoulders adducted, elbows at 90 degrees, and wrist in a neutral position. The patients performed three attempts, each involving a continuous contraction for 3 s. We calculated the mean value of the three attempts to generate the results^48^.

Patient global impression of change scale indicates the patients’ perception of change after treatment for chronic pain. The scale presents 7 items that describe a general evaluation of the patient about the treatment, such as daily activities limitations, symptoms, emotions and quality of life. They have to choose one of the following options: very much improved, much improved, minimally improved, no change, worse, much worse, or very much worse^49^.

tDCS was rigorously monitored throughout the study. Adverse events and side effects were recorded during each session and follow-up period. Patients were specifically questioned about common tDCS-related adverse events and side effects, including skin redness, headache, dizziness, and any other unexpected sensations^50^. The incidence, duration, and severity of these events were documented, and their potential association with tDCS was evaluated.

### Sample size

G-Power 3.1.9.2 (Franz Faul, Universitat Kiel, Germany) was used to calculate the sample size. We calculated the sample size based on a previous study for chronic pain^14,26^. We estimated an effect size of 0.25, an alpha error of 0.05, a power of 0.90, with two arms, sphericity of 1, and four measurement times for the ANOVA repeated measures, within-between interaction. This resulted in a total sample size of 30 participants. To account for potential dropouts and avoid a loss of power, we added four more patients, resulting the total of 34 patients.

The originally calculated sample size for this clinical trial was 46 participants, based on statistical power estimates and a more conservative effect size. However, due to several challenges, the study was completed with 34 participants (74% of the predefined sample size). During recruitment, we encountered unforeseen obstacles that limited our ability to reach the desired sample size. These challenges included logistical constraints, difficulties in identifying eligible candidates, and a lower-than-expected acceptance rate, resulting in fewer participants than initially planned. Despite not achieving the planned sample size, a post-hoc power analysis was conducted to evaluate the impact of the reduced sample size on the study’s ability to detect significant effects. The analysis revealed that the observed effect size between the active and control groups for VAS, considering the baseline, 1st or 2nd follow-up, was still large enough to indicate a significant difference (the observed effect size was a Cohen d = 1.13), even with the smaller sample size. This finding suggests that the evaluated treatment/intervention has a substantial impact on the analyzed outcomes and remains clinically relevant.

### Randomization, blinding, and allocation concealment

Patients were randomized in a 1:1 ratio to receive either active tDCS or sham tDCS (Fig. 3). An external research assistant generated the allocation sequence using a computer-generated randomization process, ensuring that each participant had an equal chance of being assigned to either group. After randomization, the research assistant inserted the allocation of each participant into opaque envelopes and only the researcher conducting the interventions had access to the envelopes. The allocation sequence was applied when new participants signed the informed consent form and were admitted into the study.

Both participants and researchers involved in assessments were blinded to group allocation throughout the trial. The researchers conducting the assessments (e.g., clinical evaluations, neurophysiological data collection) and the participants were kept completely unaware of the group assignments. This was achieved by separating the roles within the research team, where only one external researcher had access to the randomization codes and programming details. All interactions with participants, including assessments and follow-ups, were conducted by blind assessors to prevent any bias in data collection.

### Statistical analysis

The data were analyzed using Jamovi software (version 2.3.28). We analyzed sociodemographic data using the t-test and Chi-Square for continuous and categorical variables, respectively. The Generalized Estimating Equation (GEE) approach was performed, incorporating a random effect in the constant of the dataset and fixed effects in the group, time, and their interaction. Time, groups, and their interactions are independent factors. The factors A/C, B/C, Q/C, Chi-Square/DF, and intraclass correlation coefficient determined the need for incorporating fixed factors into the dataset’s model. VAS, fatigue, and functional tests were the dependent variables. We conducted two analyses for VAS: (1) at baseline, at the 15th session and two follow-ups; (2) at baseline, daily before each session and two follow-ups. For fatigue and functional tests (sit-to-stand, elbow flexion, Timed Up and Go, stationary walk, and manual dynamometer), the analyses were performed at baseline, at the 15th session and at the follow-up. The mean, mean difference (MD), standard deviation, standard error, 95% confidence interval, and p-value were reported. Bonferroni correction for multiple comparisons calculated the post-hoc comparisons. For Patient Global Impression of Change Scale, and adverse events, the Chi-squared test analyzed the frequency of distribution between the groups after the last session and at the follow-ups. The Chi-squared test analyzed the blinding effectiveness after the last intervention. The significance level for all statistical tests was set at a p-value of less than 0.05. Data imputation for one patient who died at follow-up involved replacing a missing value with the mean from the group considering all evaluation time, including baseline.

## Data Availability

The datasets generated and analyzed during the current study are available from the corresponding author on reasonable request.
